# C-reactive protein levels are associated with early cardiac complications or death in patients with acute ischemic stroke: a propensity-matched analysis of a global federated health from the TriNetX network

**DOI:** 10.1007/s11739-023-03280-1

**Published:** 2023-04-29

**Authors:** Tommaso Bucci, Dimitrios Sagris, Stephanie L. Harrison, Paula Underhill, Daniele Pastori, George Ntaios, Garry McDowell, Benjamin J. R. Buckley, Gregory Y. H. Lip

**Affiliations:** 1 Liverpool Centre for Cardiovascular Science at University of Liverpool, Liverpool John Moores University and Liverpool and Heart and Chest Hospital, William Henry Duncan Building 6 West Derby Street, Liverpool, L7 8TX UK; 2 Department of General Surgery and Surgical Specialties “Paride Stefanini”, Sapienza University of Rome, Rome, Italy; 3 Department of Internal Medicine, Faculty of Medicine, School of Health Sciences, University of Thessaly, Larissa, Greece; 4 TriNetX LLC, London, UK; 5 Department of Clinical Internal, Anesthesiological and Cardiovascular Sciences, Sapienza University of Rome, Rome, Italy; 6 Department of Clinical Medicine, Aalborg University, Aalborg, Denmark

**Keywords:** Stroke, Cardiovascular complications, Stroke–heart syndrome

## Abstract

The role of inflammation in predicting early cardiac complications among stroke patients is unclear. Electronic medical records from TriNetX, a global federated health research network, were used for this retrospective analysis. Patients with ischemic stroke and C-Reactive Protein (CRP) levels measured within 24 h post-stroke were categorized into three groups: (i) < 1 mg/L, (ii)1–3 mg/L and (iii) > 3 mg/L. The primary outcome was a composite outcome of cardiac complications (heart failure (HF), ischemic heart disease, atrial fibrillation (AF), ventricular arrhythmias and Takotsubo cardiomyopathy) or death at 30 days from the index event. Cox-regression analyses were used to produce hazard ratios (HRs) and 95% confidence intervals (CI) following 1:1 propensity score matching (PSM). Of the 104,741 patients enrolled, 51% were female and the mean age was 66 ± 16 years. After PSM, a new cardiac complication or death within 30 days occurred in 5624 (33.1%) patients with CRP > 3 mg/L, in 4243 (25.6%) patients with CRP 1–3 mg/L and in 3891 (23.5%) patients with CRP < 1 mg/L. Patients with CRP levels of 1–3 mg/L and > 3 mg/L had higher risk of the composite outcome (HR 1.10, 95%CI 1.05–1.52; HR 1.51, 95%CI 1.45–1.58), death (HR 1.43, 95%CI 1.24–1.64; HR 3.50, 95%CI 3.01–3.96), HF (HR 1.08, 95%CI 1.01–1.16; HR 1.51, 95%CI 1.41–1.61), AF (HR 1.10, 95% CI:1.02–1.18; HR 1.42, 95%CI 1.33–1.52) and ventricular arrhythmias (HR 1.25, 95%CI 1.02–1.52; HR 1.67, 95% CI 1.38–2.01) compared to those with CRP < 1 mg/L. Ischemic heart disease were more common among patients with CRP levels > 3 mg/L compared to those with CRP < 1 mg/L (HR:1.33, 95% CI:1.26–1.40), while no association with Takotsubo cardiomyopathy was found in all the analyses. CRP levels within the first 24 h of an ischemic stroke predict 30-day cardiac complications or death.

**Supplementary Information:**

The online version contains supplementary material available at 10.1007/s11739-023-03280-1.

## Introduction

During the early period following ischemic stroke, patients are exposed to a high risk of stroke recurrence and other complications [1, 2]. Among these, early cardiac complications can occur in almost 25% of patients with ischemic stroke, a finding which was associated with stroke recurrence in more than half of these patients and twofold increase in the risk of 5-year mortality [3]. Such post-stroke early cardiac complications, which can be collectively described as the stroke–heart Syndrome, include ventricular dysrhythmias, heart failure (HF), atrial fibrillation/flutter (AF), ischemic heart disease and Takotsubo cardiomyopathy [3, 4]. Stroke–heart syndrome (SHS) represents a combination of local and distal inflammatory and neurogenic dysregulations, which may lead to cardiac dysfunction and clinically relevant cardiac events [5]. Although the risk of early cardiac complications is higher among patients with cardiovascular risk factors [6], these complex pathophysiological processes may lead to stroke–heart syndrome in patients with and without underlying heart disease, as a result of catecholamine release and the upregulation of pro-inflammatory cytokines [5, 7]. Among these pro-inflammatory mediators, interleukin-1 (IL-1) and IL-6 have been correlated with local and systemic complications such as myocardial damage, being potential target-molecules in acute stroke treatment [8, 9]. As downstream biomarkers of IL-1 induction, elevated plasma levels of C-reactive protein (CRP) and IL-6 may be predictive of poor clinical outcome in the acute phase of ischemic stroke [10–12]. Accordingly, two phase-II randomized clinical trials using IL-1 receptor antagonists in acute stroke, confirmed that IL-1 inhibition was associated with a significant reduction in CRP levels [13, 14]. To correlate inflammatory biomarkers with cardiovascular outcomes, the American Heart Association proposed cardiovascular risk stratification based on CRP levels, as a surrogate marker of systemic vascular inflammatory processes [15]. Although post-stroke CRP levels have been previously associated with long-term outcomes [16, 17], CRP levels and the relationship with early cardiac complications (i.e., SHS) have not been previously investigated.

The aim of this study is to evaluate the association of CRP levels during the first 24 h after ischemic stroke with the risk of SHS during the first 30 days after the ischemic stroke, in a real-world, global federated health network.

## Methods

### Study design

This was a retrospective observational study conducted within TriNetX, a global federated health research network with access to electronic medical records (EMRs) from participating health care organizations including academic medical centers, specialty physician practices, and community hospitals covering approximately 69.8 million individuals, mainly in the United States. Within this network, available data include demographics, diagnoses using International Classification of Diseases, Ninth Revision and Tenth Revision, Clinical Modification (ICD-10-CM) codes, and medications. More information can be found online (https://trinetx.com/company‐overview/).

### Cohort

The searches on the TriNetX online research platform were performed on the 1^st^ of February 2023 for individuals aged ≥ 18 years with ischemic stroke (termed as Cerebral infarction ICD-10-CM code I63) and CRP measurement on admission and during the first 24 h from stroke, recorded in electronic medical records. To include the highest number of patients possible, the searches were not restricted to a specific time period; however, more than 95% of patients considered in this study were entered in the TriNetX platform between 2010 and 2020. The choice to consider the first 24 h after the admission was made to make homogeneous the different time of CRP collection.

At the time of the search, 65 participating health care organizations had data available for patients who met the study inclusion criteria. The baseline index event date was the date of ischemic stroke, while any diagnoses registered before this date were the individual’s baseline characteristics.

The cohort was divided into groups using electronic health records according to CRP levels during the first 24 h after the ischemic stroke, based on the suggested cut-off thresholds of the American Heart Association statement of cardiovascular risk stratification, in three groups: (i) those with CRP levels < 1 mg/L, (ii) those with CRP levels 1–3 mg/L, and (iii) with CRP levels > 3 mg/L [15].

### Outcomes

The primary outcome was the diagnosis of the composite outcome of early cardiac complications or death within 30 days after the ischemic stroke, comprised of death, ischemic heart disease, HF, AF, ventricular arrhythmias and Takotsubo cardiomyopathy. Secondary outcomes were the risk for each component of the composite primary outcome within 30 days after the ischemic stroke. The occurrence of the primary and secondary outcomes was analyzed based on the levels of CRP during the first 24 h after the index ischemic stroke. The cardiac complications of interest within 30 days of ischemic stroke were identified via ICD-10-CM code. (Supplementary Table 1).

To estimate the risk of a new cardiac post-stroke complications, we prespecified an exploratory analysis excluding for every outcome of interest those patients who experienced a similar outcome before the index event.

Additionally, to test the generalizability of our hypothesis, we further investigated the risk of the composite outcome in two different sensitivity analyses: (i) in elderly patients with age > 65 years, and (ii) patients without possible confounding factors such as systemic connective tissue disease and recent (1 month before) diagnosis of sepsis or pneumonia and glucocorticoids or antibiotics use.

### Statistical analysis

All statistical analyses were performed on the TriNetX online research platform. Baseline characteristics were compared using chi-squared tests for categorical variables and independent-sample t-tests for continuous variables. We performed 1:1 propensity score matching (PSM) to create balanced cohorts. We included the following variables in the PSM: age, sex, ethnicity, arterial hypertension, ischemic heart diseases, ischemic stroke, HF, pulmonary heart disease/disease of the pulmonary circulation, diabetes, peripheral arterial disease, cardiovascular procedures (including electrocardiography, echocardiography, catheterization, cardiac devices, and electrophysiological procedure), respiratory infection (pneumonia), sepsis, systemic connective tissue disease (Vasculitis, Systemic Lupus Erythematosus, Dermatopolyositis, Systemic Sclerosis, Sjögren syndrome, Behçet’s disease, Polymyalgia Rheumatica, Multisystem Inflammatory Syndrome) and cardiovascular medications (including anticoagulants, antiplatelets, β-blockers, antiarrhythmics, diuretics, antilipemic agents, antianginals, calcium channel blockers, and angiotensin-converting enzyme inhibitors and angiotensin II inhibitors). After PSM, we used Cox proportional hazard models to assess the association between CRP levels and cardiac complications/death. Participants were censored when they experienced the outcome of interest or when they died.

Additionally, sensitivity analyses were performed to investigate the association of CRP levels with early cardiac complications or death in ischemic stroke patients age ≥ 65 years and excluding patients with systemic connective tissue disease, recent (within 1 month) diagnosis of sepsis, pneumonia or glucocorticoids and antibiotics in order to reduce the possibility of confounding factors that may increase CRP. In this analysis, Cox-regression proportional hazard model was used to calculate hazard ratios (HRs) and 95% confidence intervals (Cis). All tests were two tailed and p-values of ≤ 0.05 were taken to indicate statistical significance. All analyses were performed in the TriNetX platform which uses R’s survival package v3.2-3.

### Data availability statement and ethical approval

TriNetx is a research network utilized for several scientific purposes, compliant with the Health Insurance Portability and Accountability Act and the US federal law which protects the privacy and security of healthcare data, including de-identified data as per the de-identification standard of the HIPAA Privacy Rule(https://trinetx.com/real-world-resources/publications/). To gain access to the data in the TriNetX research network, requests are directed to TriNetX and a data sharing agreement is required. As a federated research network, studies using the TriNetX health research network do not need ethical approval as no patient identifiable identification is received. Further information about the data extraction from TriNetX is reported in the supplementary material.

## Results

The initial cohort consisted of 104,741 patients with ischemic stroke (mean age 65.8 ± 16.4, 51.4% female) and CRP measured during the first 24 h from the index event from 65 (primarily United States) health care organization with mean age of 65.8 ± 16.4 (51.4% female). Of these, 67,047 (64.0%) had hypertension, 35,202 (33.6%) coronary artery disease, 38,567 (36.8%) diabetes mellitus, 20,748 (19.8%) AF and 23,537 (22.5%) HF. Among patients with available CRP measurement within 24 h after the ischemic stroke, 17,044 (16.2%) patients had CRP levels < 1 mg/L, 21,643 (20.6%) had CRP levels of 1–3 mg/L and 66,054 (63.2%) had CRP levels of > 3 mg/L. In the initial cohort patients with higher CRP levels had significantly more frequently cardiovascular comorbidities and infections (i.e., pneumonia and sepsis) and were treated more frequently with cardiovascular medication (Supplementary Table 2 and 3).

Before PSM, patients with CRP 1–3 mg/L and > 3 mg/L were older, with a higher prevalence of cardiovascular risk factors, infectious and systemic connective tissue disease and more treated with cardiovascular medications compared to those with CRP < 1 mg/L (Supplementary Tables 2 and 3).

After PSM on a 1:1 ratio for the comparison between CRP levels < 1 mg/L and 1–3 mg/L, the cohort consisted of 33,138 patients (mean age 64.9 ± 16.9 years; 18,587 (54.7%) female), while for the comparison between CRP levels < 1 mg/L and > 3 mg/L, the cohort consisted of 34,024 patients (mean age 64.5 ± 17.2 years; 18,587 (54.7%) female) (Table 1). After PSM, both the comparisons were well matched for age, sex, ethnicity, comorbidities and cardiovascular treatment (Table 1).

**Table 1:** Baseline characteristics of ischemic stroke patients based on C-reactive protein levels after propensity score matching

	< 1 mg/L *n* = 16,569		1–3 mg/L *n* = 16,569		*p*-value	< 1 mg/L *n* = 17,012		> 3 mg/L *n* = 17,012		*p*-value
Demographics *n* (%)										
Age, years (± SD)	64.9 (16.8)		64.9 (17.1)		0.948	64.5 (17.1)		64.7 (17.2)		0.237
White	11,932 (72.0)		11,928 (72.0)		0.961	12,297 (72.3)		12,307 (72.3)		0.904
Black	2546 (15.4)		2504 (15.1)		0.521	2570 (15.1)		2647 (15.6)		0.247
Asian	402 (2.4)		403 (2.4)		0.972	455 (2.7)		460 (2.7)		0.867
Female	9281 (54.6)		9306 (54.7)		0.785	9281 (54.6)		9306 (54.7)		0.785
Comorbidities *n* (%)										
Heart failure	2681 (16.2)		2643 (16.0)		0.570	2700 (15.9)		2749 (16.2)		0.469
Arterial hypertension	9578 (57.8)		9509 (57.4)		0.443	9744 (57.3)		9762 (57.4)		0.844
Ischemic heart disease	4790 (28.9)		4730 (28.5)		0.466	4895 (28.8)		4927 (29.0)		0.702
Pulmonary heart disease and diseases of pulmonary circulation	1639 (9.9)		1608 (9.7)		0.567	1669 (9.8)		1744 (10.3)		0.176
Atrial Fibrillation	2372 (14.3)		2369 (14.3)		0.962	2375 (14.0)		2426 (14.3)		0.427
Ischemic stroke	9798 (59.1)		9773 (59.0)		0.780	9941 (58.4)		10,006 (58.8)		0.474
Dyslipidemia	7445 (44.9)		7434 (44.9)		0.903	7582 (44.6)		7587 (44.6)		0.957
Diabetes mellitus	5473 (33.0)		5386 (32.5)		0.309	5616 (33.0)		5599 (32.9)		0.845
Obesity	3676 (22.2)		3692 (22.3)		0.833	3769 (22.2)		3897 (22.9)		0.097
Chronic kidney disease	3373 (20.4)		3320 (20.0)		0.468	3544 (20.8)		3525 (20.7)		0.800
Pneumonia	2557 (15.4)		2539 (15.3)		0.784	2636 (15.5)		2640 (15.5)		0.952
Sepsis	1446 (8.7)		1419 (8.6)		0.598	1463 (8.6)		1512 (8.9)		0.347
Peripheral arterial disease	1914 (11.6)		1912 (11.5)		0.973	1966 (11.6)		2048 (12.0)		0.168
Systemic connective disorders	2383 (14.4)		2385 (14.4)		0.975	2565 (15.1)		2641 (15.5)		0.252
Procedures *n* (%)										
Electrocardiography	8329 (50.3)		8286 (50.0)		0.637	8570 (50.4)		8558 (50.3)		0.896
Echocardiography	5083 (30.7)		5052 (30.5)		0.712	5192 (30.5)		5230 (30.7)		0.655
Cardiac device	481 (2.9)		479 (2.9)		0.807	483 (2.8)		542 (3.2)		0.547
Electrophysiological procedures	160 (1.0)		165 (1.0)		0.780	162 (1.0)		175 (1.0)		0.477
Cardiac catheterization procedures	891 (5.4)		881 (5.3)		0.807	894 (5.3)		954 (5.6)		0.151
Concomitant medications *n* (%)										
Lipid-lowering treatment	7848 (47.4)		7842 (47.3)		0.947	7937 (46.7)		7915 (46.5)		0.811
Beta-blockers	6749 (40.7)		6704 (40.5)		0.615	6822 (40.1)		6825 (40.1)		0.974
Diuretics	5520 (33.3)		5487 (33.1)		0.700	5609 (33.0)		5560 (32.7)		0.572
Antiarrhythmics	7052 (42.6)		7054 (42.6)		0.982	7308 (43.0)		7302 (42.9)		0.948
Calcium channel blockers	5046 (30.5)		5042 (30.4)		0.962	5115 (32.1)		5139 (30.2)		0.777
ACE inhibitors	4410 (26.5)		4402 (26.6)		0.735	4460 (26.2)		4556 (26.8)		0.238
Angiotensin II inhibitors	2610 (15.8)		2613 (15.8)		0.850	2655 (15.6)		2595 (15.3)		0.705
Antianginals	1971 (13.0)		1972 (13.0)		0.986	2210 (13.0)		2290 (13.5)		0.200
Anticoagulant therapy	2186 (13.2)		2172 (13.1)		0.530	7756 (45.6)		7672 (45.1)		0.360
Antiplatelet therapy	8617 (52.0)		8599 (51.9)		0.843	8778 (51.6)		8777 (51.6)		0.991

*CRP* C-reactive protein, *SD* standard deviation, *ACE* angiotensin-converting enzyme

### Risk of cardiovascular complications comparing patients with CRP 1–3 vs < 1 mg/L

After PSM, among 33,138 patients, the primary composite outcome of cardiac complications or death within 30 days of the index ischemic stroke were reported in 4243 (25.6%) patients with CRP levels 1–3 mg/L compared to 3891 (23.5%) among those with CRP levels < 1 mg/L (HR 1.10, 95% CI 1.05–1.15).

Analyzing the risk for secondary outcomes (each component of the composite primary outcome), patients with CRP levels of 1–3 mg/L showed a higher risk death (HR:1.43, 95% CI:1.24–1.64), HF (HR 1.08, 95% CI 1.01–1.16), AF (HR 1.10, 95% CI 1.02–1.18) and ventricular arrhythmias (HR 1.25, 95%CI 1.02–1.52), compared to those with CRP levels of < 1 mg/L. In patients with CRP levels 1–3 mg/L within the first 24 h of stroke, no significant increased risk of ischemic heart disease (HR 1.05, 95%CI 0.99–1.11) or Takotsubo cardiomyopathy (HR 0.95, 95%CI 0.53–1.71) was found when compared to those with CRP levels < 1 mg/L.

### Exploratory analysis for the risk of new-onset cardiac complications.

In the exploratory analysis after excluding patients who had experienced the cardiovascular outcome of interest before the index event (i.e., focusing on *new onset* cardiac complications), the primary composite outcome of new cardiac complications or death occurred in 7.6% of patients with CRP levels 1–3 mg/L compared to 6.4% among those with CRP levels < 1 mg/L (HR 1.18, 95% CI 1.06–1.32). However, analyzing the risk for secondary outcomes, patients with CRP levels 1–3 mg/L showed no significative increased risk of ischemic heart disease (HR 1.06, 95%CI 0.91–1.24), HF (HR 1.15, 95%CI 0.97–1.36), AF (HR:1.12, 95%CI:0.96–1.32), ventricular arrhythmias (HR 1.08, 95%CI 0.77–1.52) and Takotsubo cardiomyopathy (HR 1.16, 95% CI 0.39–3.45) compared to those with CRP levels < 1 mg/L.

*Risk of cardiovascular complications* comparing patients with CRP > 3 vs < 1 mg/L.

After PSM, among 34,024 patients, cardiac complications or death within 30 days of the index ischemic stroke occurred in 5624 (33.1%) patients with CRP levels > 3 mg/L compared to 3947 (23.2%) in those with CRP levels < 1 mg/L (HR 1.51, 95% CI 1.45–1.58).

**Fig. 1:**
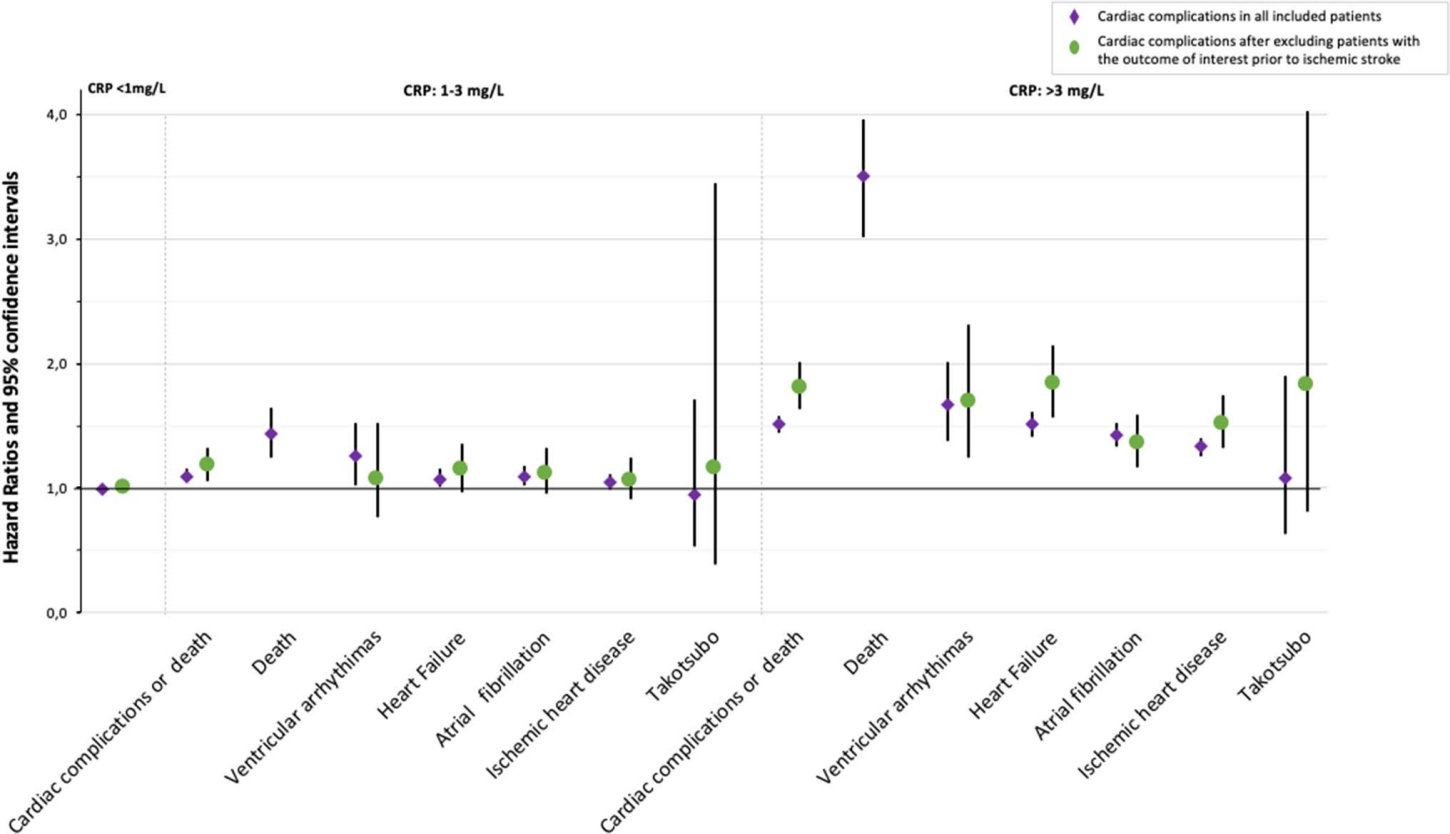
Relative hazard ratios and 95% confidence intervals for each outcome related to C-reactive protein (CRP) levels

Analyzing the risk for secondary outcomes, CRP levels of > 3 mg/L were associated with significantly higher risk of death (HR:3.50, 95% CI:3.01–3.96), ischemic heart disease (HR 1.33, 95% CI 1.26–1.40), HF (HR 1.51, 95% CI 1.41–1.61), AF (HR 1.42, 95% CI 1.33–1.52) and ventricular arrhythmias (HR 1.67, 95% CI 1.38–2.01) compared to those with CRP levels of < 1 mg/L (Fig. 1). No significant increased risk of Takotsubo cardiomyopathy was found in patients with CRP levels > 3 mg/L within the first 24 h of stroke compared to those with CRP levels < 1 mg/L (HR 1.09, 95% CI 0.63–1.90).

### Exploratory analysis for the risk of new-onset cardiac complications.

In the exploratory analysis after excluding patients who had experienced the cardiovascular outcome of interest before the index ischemic stroke, the primary composite outcome of new cardiac complications or death occurred in 11.4% of patients with CRP levels > 3 mg/L compared to 6.4% among those with CRP levels < 1 mg/L (HR 1.81, 95% CI 1.63–2.01).

For secondary outcomes, in patients with CRP levels > 3 mg/L the significative higher risk for ischemic heart disease (HR 1.52, 95% CI 1.32–1.75), HF (HR 1.84, 95% CI 1.57–2.15), AF (HR 1.36, 95% CI 1.17–1.59) and ventricular arrhythmias (HR 1.69, 95% CI 1.24–2.31) compared to those with CRP levels < 1 mg/L remained consistent, as well as the lack of any association with Takotsubo cardiomyopathy (HR:1.83, 95% CI:0.68–4.96) (Fig. 1).

### Sensitivity analyses

In our study, the mean age of patients with stroke was 65.8 ± 16.4 years, making difficult to generalize our findings in elderly. To investigate the association of CRP levels with cardiac complications or death after stroke in the elderly, we performed a sensitivity analysis including only > 65 years old. Among the 63,492 identified elderly patients (mean age 78.0 ± 8.1 years, 51% females) with available CRP measurement within 24 h after the ischemic stroke, 9,249 (16.2%) had CRP levels < 1 mg/L, 12,586 (20.6%) CRP levels of 1–3 mg/L, and 41,657 (63.2%) CRP levels of > 3 mg/L. Characteristics of the cohort population based on the levels of CRP before and after PSM can be found in Supplementary Tables 4 and 5. After PSM, among ischemic stroke patients > 65 years old, CRP levels 1–3 mg/L and > 3 mg/L, were associated with higher risk of early cardiac complications or death (HR 1.11, 95% CI 1.05–1.17 and HR 1.44, 95% CI 1.37–1.51, respectively).

In the sensitivity analysis after excluding patients with systemic connective tissue disease and with recent (within 1 month) diagnosis of sepsis, pneumonia or glucocorticoids and antibiotics use, we identified 32,644 patients (mean age 68.0 ± 16.1 years, 48.9% females). Among them, 6546 (20.1%) had CRP levels < 1 mg/L, 8458 (25.9%) had CRP levels of 1–3 mg/L and 17,640 (54.0%) CRP levels > 3 mg/L. Baseline characteristics before and after PSM can be found in Supplementary Table 6 and 7. After PSM, among ischemic stroke patients without connective tissue disorders, recent infections or associated treatments (glucocorticosteroids/antibiotics), CRP levels 1–3 mg/L and > 3 mg/L, were associated with higher risk of early cardiac complications or death (HR 1.13, 95% CI 1.04–1.23 and HR 1.40, 95% CI 1.30–1.52, respectively).

## Discussion

The results from this large multicenter cohort study indicate that increased CRP levels during the first 24 h of hospitalization for ischemic stroke were associated with the occurrence of early cardiac complications, suggesting that CRP may be a possible biomarker for stroke–heart syndrome. There appears to be a dose response with this early risk, as evident by increasing risk of stroke–heart syndrome and death from CRP 1–3 mg/L to CRP > 3 mg/L.

CRP levels during the first 24 h have been previously associated with significantly higher risk of death or new cardiovascular event during the first year after acute ischemic stroke [17]. Similarly, a recent study showed that higher CRP levels in the early post-stroke phase up to 72 h, were associated with increased risk of stroke recurrence at 1 year [16]. Nevertheless, these small sample size studies investigated the association of CRP with one-year cardiovascular outcomes and not early cardiac complications. Early cardiac complications were previously found to be more common in patients with severe strokes with medical history of HF, diabetes, higher creatinine levels, and ECG abnormalities; however, the association of CRP levels with early cardiac complications was not investigated [6].

In the present study, compared to patients with CRP < 1 mg/dL, early cardiac abnormalities; HF, AF and new episodes of ventricular arrhythmias were more frequent in patients with a relatively small increase in CRP levels (1–3 mg/L), while, ischemic heart disease or Takotsubo cardiomyopathy were not associated with this increased level of CRP. Among those with a higher increased CRP level (> 3 mg/L), new cardiac complications; ischemic heart disease, HF, AF and ventricular arrhythmias, were more common than among patients with CRP levels < 1 mg/L, while Takotsubo cardiomyopathy was not statistically associated with this CRP level.

Whether the association of increased CRP with new cardiac complications represents a pathophysiological mechanism or a biomarker of high concomitant atherosclerotic burden among stroke patients is unclear. Patients with ischemic stroke are at very high risk of cardiovascular events, associated with higher atherothrombotic burden and cardiovascular risk factors [18, 19]. Among these patients, inflammatory mediators such as IL-1, IL-6 and CRP are highly associated with the atherothrombotic burden [9]. In this study, only CRP levels of > 3 mg/L were associated with new onset ischemic heart disease, potentially representing the pre-stroke atherothrombotic burden and vascular inflammation. Similarly, Takotsubo cardiomyopathy, which represents the acute dysfunction of a previously untouched myocardium associated with the overexpression of inflammatory mediators [20, 21], was not associated with increased levels of CRP. This could be explained by the fact that the included patients were well balanced for the diagnosis of sepsis, which represents an important cause of Takotsubo cardiomyopathy, associated with the overexpression of hyperinflammatory mediators [22].

On the other hand, we cannot exclude the possibility that this CRP elevation was associated directly to stroke and the release of IL-1β both in the brain and in the systemic circulation, inducing a cascade of sympathetic system activation catecholamine release in the extracellular myocardium [5, 7]. Irrespective of whether CRP is pathophysiologically associated with cardiac complications or represents a biomarker of undiagnosed atherosclerotic burden, among patients with ischemic stroke, CRP was predictive of 30-day cardiac complications or death, irrespective of the presence or absence of previous cardiac diseases.

### Strengths and limitations

This is the first study to provide evidence of an association between elevated CRP levels during the first 24 h after stroke 30-day cardiac complications/death. CRP is a low-cost and easy-to-measure biomarker in clinical practice. Due to its low-cost and standardized measurement, CRP measurement can be used universally, even in settings of restricted resources and provide valuable information for the treating physician to identify those at high-risk of major cardiac complication related to ischemic stroke. Based on our expanding knowledge in cardiovascular inflammation and the use of immunomodulatory agents [23], the identification of patients with high inflammatory and atherosclerotic burden could generate the hypothesis that biomarkers such as CRP, could potentially guide the use of immunomodulatory agents, to prevent post-stroke cardiac complications. Nonetheless, several limitations of this analysis are noteworthy. The major limitation of the study is its retrospective nature based on Health care organization EMR data which are subject to entry errors and data gaps, while outcomes which occurred outside the TriNetX network may have not been well captured. Furthermore, in our analysis we included patients who had CRP values captured on admission or during the first 24 h, but we could not further stratify our patients based on the time of blood sample collection or the dynamic changes of CRP levels during the first 24 h. All these factors did not allow as to further investigate the association of CRP level changes with the development of cardiovascular complications or all-cause mortality. Additionally, we were not able to determine the impact or match the included ischemic stroke patients based on stroke severity using the NIHSS or stroke volume of the index event since these data were not available in the TriNetX platform. Moreover, we could not balance cohorts based on hyperacute revascularization therapies (i.e. thrombolysis and thrombectomy), hemorrhagic transformation of the index stroke or any discharge data since the TriNetX database does not provide ability to balance cohorts based on characteristics that were captured in the database after the index event. Additionally, the collected variables in the electronic database were not prespecified and residual confounding may have influenced some of our results, including socioeconomic status, quality of care, and risk factor control. The presence of elevated CRP among patients with ischemic stroke could be attributed to the presence of concomitant infections. For example, pneumonia or concomitant systemic autoimmune disease, especially among stroke patients, are associated with poorer outcome and higher risk of death [24]. Although this could partially explain the significantly higher risk of cardiac complications and death seen in patients with CRP levels > 3 mg/L, compared to those with CRP levels < 1 mg/L, this higher risk was further confirmed also in the sensitivity analysis excluding patients with pneumonia, sepsis, and connective tissue disease or who utilized antibiotics or steroids at least one month before the index event. Still, irrespective of the presence of concomitant infection, systemic autoimmune disease or the presence of atherosclerotic burden, CRP elevation was predictive of the SHS.

## Conclusion

In conclusion, CRP levels in the first 24 h of ischemic stroke are associated with cardiac complications or death within 30 days after stroke. This accessible and low-cost biomarker could be used to identify high-risk patients and predict complications of the stroke–heart syndrome.

## Supplementary Information

Below is the link to the electronic supplementary material.

Supplementary file1 (DOCX 120 KB)

## Data Availability

Data will be made available on request.
