# Supplementary material for: C-reactive protein levels are associated with early cardiac complications or death in patients with acute ischemic stroke: a propensity-matched analysis of a global federated health from the TriNetX network
Source: Intern Emerg Med. 2023 Apr 29;18(5):1329–36. doi: 10.1007/s11739-023-03280-1 (PMC10412660; doi:10.1007/s11739-023-03280-1)
Supplement: Supplementary file 1 — Supplementary file1 (DOCX 120 KB) [file 11739_2023_3280_MOESM1_ESM.docx]

**SUPPLEMENTARY DATA**

**Supplementary detail regarding methods**

TriNetX Database

The TriNetX data are collected from member healthcare organizations (HCO) and originates from their primary electronic health records (EHR) system. A typical HCO is a large academic health center with data coming from majority of its affiliates. A single HCO frequently has more than one facility, including main and satellite hospitals. The data are stored on the TriNetX database via a physical server at the institution’s data centre or a virtual hosted appliance. The TriNetX platform comprises of a series of these appliances connected into a federated network. This network can broadcast queries to each appliance. Results are subsequently collected and aggregated. Once the data are sent to the network, it is mapped to a standard and controlled set of clinical terminologies and undergoes a data quality assessment including ‘data cleaning’ that rejects records which do not meet the TriNetX quality standards. The TriNetX database performs internal and extensive data quality assessment with every refresh based on conformance, completeness, and plausibility (http://doi.org/10.13063/2327-9214.1244). HIPAA (Health Insurance Portability and Accountability Act) compliance of the clinical patient data is achieved using deidentification. Available data types within the network include demographics, diagnoses (represented by ICD-10-CM codes), procedures (coded in ICD-10-PCS or CPT), and measurements (coded to LOINC). While extensive information is provided about patients’ diagnoses and procedures, other variables (such as socioeconomic and lifetime factors are not comprehensively represented). The advantage of electronic health record data over insurance claim data is that both insured and uninsured patients are included. An advantage of electronic health record data over survey data is that the former represents the diagnostic rates in the population presenting to healthcare facilities. This provides an accurate account of the burden of specific diagnoses on healthcare systems. One primary limitation of relying on diagnoses is that they do not account for undiagnosed patients who might have a condition but have not yet received medical support. Another general limitation of electronic health record data is that a patient may be seen in different HCO for different components of their care. If one healthcare organization is not part of the federated network, then part of their medical records may not be available. Using a network of healthcare organizations, rather than a single site, limits this possibility but does not fully remove it. Propensity Score Matched Analyses Using logistic regression [Logistic Regression of the scikit-learn package in Python (version 3.7)], TriNetX performs a 1:1 greedy nearest neighbor matching model, with a caliper of 0.1 pooled standard deviations. To eliminate bias resulting from nearest neighbour algorithms, the orders of rows are randomized. Any baseline characteristic with a standardised mean difference between cohorts lower than 0.1 is deemed well matched (https://www.tandfonline.com/doi/full/10.1080/00273171.2011.568786). To prevent inadvertent disclosure of protected health information, patient counts for demographics, clinical characteristics, and outcomes of less than 10 are reported as ≤10

**Supplementary Table 1.** ICD-10-CM codes for early cardiovascular complications (within 4-weeks of an ischemic stroke) and 5-year mortality.

| **Early cardiovascular complications** | **ICD-10-CM-codes** |
| --- | --- |
| Ischemic heart disease | The composite of any of the following:   - I20.0 [unstable angina] - I21 [acute myocardial infarction] - I22 [subsequent ST elevation and non-ST elevation myocardial infarction] - I23 [Certain current complications following ST elevation (STEMI) and non-ST elevation (NSTEMI) myocardial infarction] - I24 [Other acute ischemic heart diseases] - I25 [Chronic ischemic heart disease] |
| AF | - I48 [Atrial fibrillation and flutter |
| Ventricular arrhythmias | - I49.0 Ventricular fibrillation and flutter   and/or   - I47.2 Ventricular tachycardia |
| Heart Failure | - I50 [Heart failure] |
| Takotsubo cardiopathy | - I51.81 [Takotsubo syndrome] |

**Supplementary Table 2.** Baseline characteristics comparison, before propensity score matching, of ischemic stroke patients with C-Reactive Protein levels 1-3 mg/L (cohort 1) and <1 mg/L (cohort 2).

| **Cohort 1 (N = 21,643) and cohort 2 (N = 17,044)** | | | | | | | | | |
| --- | --- | --- | --- | --- | --- | --- | --- | --- | --- |
|  | **Demographics** | | | | | | | | |
|  |  | Cohort | |  | Mean ± SD | Patients | % of Cohort | p-Value | Std diff. |
|  |  | 1 2 | Age | Age | 66.4 +/- 16.7 64.5 +/- 17.1 | 21,609 17,023 | 100% 100% | <0.001 | 0.114 |
|  |  | 1 2 | 2106-3 | White |  | 14,869 12,302 | 68.8% 72.3% | <0.001 | 0.076 |
|  |  | 1 2 | F | Female |  | 11,306 9,290 | 52.3% 54.6% | <0.001 | 0.045 |
|  |  | 1 2 | 2054-5 | Black or African American |  | 3,543 2,570 | 16.4% 15.1% | 0.001 | 0.036 |
|  |  | 1 2 | 2028-9 | Asian |  | 431 461 | 2.0% 2.7% | <0.001 | 0.047 |
|  | **Diagnosis** | | | | | | | | |
|  |  | Cohort | |  | Mean ± SD | Patients | % of Cohort | P-Value | Std diff. |
|  |  | 1 2 | I10-I16 | Arterial hypertension |  | 13,244 9,747 | 61.3% 57.3% | <0.001 | 0.082 |
|  |  | 1 2 | I20-I25 | Ischemic heart diseases |  | 6,563 4,900 | 30.4% 28.8% | 0.001 | 0.035 |
|  |  | 1 2 | I48 | Atrial fibrillation and flutter |  | 3,668 2,376 | 17.0% 14.0% | <0.001 | 0.084 |
|  |  | 1 2 | I50 | Heart failure |  | 3,946 2,700 | 18.3% 15.9% | <0.001 | 0.064 |
|  |  | 1 2 | I26-I28 | Pulmonary heart disease and diseases of pulmonary circulation |  | 2,278 1,669 | 10.5% 9.8% | 0.017 | 0.024 |
|  |  | 1 2 | E78 | Dyslipidaemia |  | 10,198 7,589 | 47.2% 44.6% | <0.001 | 0.052 |
|  |  | 1 2 | E08-E13 | Diabetes mellitus |  | 7,323 5,621 | 33.9% 33.0% | 0.073 | 0.018 |
|  |  | 1 2 | E65-E68 | Obesity |  | 4,915 3,772 | 22.7% 22.2% | 0.170 | 0.014 |
|  |  | 1 2 | N18 | Chronic kidney disease (CKD) |  | 4,235 3,548 | 19.6% 20.8% | 0.002 | 0.031 |
|  |  | 1 2 | I63 | Cerebral infarction |  | 13,569 9,950 | 62.8% 58.5% | <0.001 | 0.089 |
|  |  | 1 2 | I73.9 | Peripheral arterial disease |  | 2,496 1,967 | 11.6% 11.6% | 0.990 | <0.001 |
|  |  | 1 2 | J18 | Pneumonia |  | 3,364 2,639 | 15.6% 15.5% | 0.861 | 0.002 |
|  |  | 1 2 | A41 | Sepsis |  | 2,044 1,463 | 9.5% 8.6% | 0.003 | 0.030 |
|  |  | 1 2 | M30-M36 | Systemic connective tissue disorders |  | 2,744 2,575 | 12.7% 15.1% | <0.001 | 0.070 |
|  | **Procedure** | | | | | | | | |
|  |  | Cohort | |  | Mean ± SD | Patients | % of Cohort | p-Value | Std diff. |
|  |  | 1 2 | 1013050 | Echocardiography Procedures |  | 6,844 5,196 | 31.7% 30.5% | 0.016 | 0.025 |
|  |  | 1 2 | 1013071 | Cardiac Catheterization Procedures |  | 1,310 894 | 6.1% 5.3% | 0.001 | 0.035 |
|  |  | 1 2 | 1019075 | Implantable, Insertable, and Wearable Cardiac Device Evaluations |  | 703 484 | 3.3% 2.8% | 0.020 | 0.024 |
|  |  | 1 2 | 1013109 | Intracardiac Electrophysiological Procedures/Studies |  | 257 162 | 1.2% 1.0% | 0.025 | 0.023 |
|  |  | 1 2 | 1013012 | Electrocardiogram, routine ECG with at least 12 leads |  | 10,989 8,575 | 50.9% 50.4% | 0.348 | 0.010 |
|  | **Medication** | | | | | | | | |
|  |  | Cohort | |  | Mean ± SD | Patients | % of Cohort | P-Value | Std diff. |
|  |  | 1 2 | CV350 | Antilipemic agents |  | 11,004 7,940 | 50.9% 46.6% | <0.001 | 0.086 |
|  |  | 1 2 | CV100 | Beta blockers/related |  | 9,629 6,824 | 44.6% 40.1% | <0.001 | 0.091 |
|  |  | 1 2 | CV300 | Antiarrhythmics |  | 9,129 7,317 | 42.2% 43.0% | 0.146 | 0.015 |
|  |  | 1 2 | CV700 | Diuretics |  | 7,753 5,609 | 35.9% 32.9% | <0.001 | 0.062 |
|  |  | 1 2 | CV200 | Calcium channel blockers |  | 7,009 5,116 | 32.4% 30.1% | <0.001 | 0.051 |
|  |  | 1 2 | CV800 | ACE inhibitors |  | 6,203 4,461 | 28.7% 26.2% | <0.001 | 0.056 |
|  |  | 1 2 | CV805 | Angiotensin II inhibitor |  | 3,625 2,656 | 16.8% 15.6% | 0.002 | 0.032 |
|  |  | 1 2 | CV250 | Antianginals |  | 3,115 2,210 | 14.4% 13.0% | <0.001 | 0.042 |
|  |  | 1 2 | BL110 | Anticoagulant therapy |  | 10,669 7,757 | 49.4% 45.6% | <0.001 | 0.076 |
|  |  | 1 2 | BL117 | Antiplatelet therapy |  | 11,821 8,784 | 54.7% 51.6% | <0.001 | 0.062 |

**Supplementary Table 3.** Baseline characteristics comparison, before propensity score matching, of ischemic stroke patients with C-Reactive Protein levels >3 mg/L (cohort 1) and <1 mg/L (cohort 2).

| **Cohort 1 (N = 66,054) and cohort 2 (N = 17,044)** | | | | | | | | | |
| --- | --- | --- | --- | --- | --- | --- | --- | --- | --- |
|  | **Demographics** | | | | | | | | |
|  |  | Cohort | |  | Mean ± SD | Patients | % of Cohort | p-Value | Std diff. |
|  |  | 1 2 | Age | Age | 68.2 +/- 16.1 64.5 +/- 17.1 | 65,910 17,023 | 100% 100% | <0.001 | 0.224 |
|  |  | 1 2 | 2106-3 | White |  | 43,574 12,302 | 66.1% 72.3% | <0.001 | 0.134 |
|  |  | 1 2 | F | Female |  | 32,763 9,290 | 49.7% 54.6% | <0.001 | 0.097 |
|  |  | 1 2 | 2054-5 | Black or African American |  | 12,639 2,570 | 19.2% 15.1% | <0.001 | 0.108 |
|  |  | 1 2 | 2028-9 | Asian |  | 968 461 | 1.5% 2.7% | <0.001 | 0.087 |
|  | **Diagnosis** | | | | | | | | |
|  |  | Cohort | |  | Mean ± SD | Patients | % of Cohort | p-Value | Std diff. |
|  |  | 1 2 | I10-I16 | Arterial hypertension |  | 44,056 9,747 | 66.8% 57.3% | <0.001 | 0.198 |
|  |  | 1 2 | I20-I25 | Ischemic heart diseases |  | 23,739 4,900 | 36.0% 28.8% | <0.001 | 0.155 |
|  |  | 1 2 | I48 | Atrial fibrillation and flutter |  | 14,684 2,376 | 22.3% 14.0% | <0.001 | 0.217 |
|  |  | 1 2 | I50 | Heart failure |  | 16,891 2,700 | 25.6% 15.9% | <0.001 | 0.243 |
|  |  | 1 2 | I26-I28 | Pulmonary heart disease and diseases of pulmonary circulation |  | 9,629 1,669 | 14.6% 9.8% | <0.001 | 0.147 |
|  |  | 1 2 | E78 | Dyslipidaemia |  | 31,746 7,589 | 48.2% 44.6% | <0.001 | 0.072 |
|  |  | 1 2 | E08-E13 | Diabetes mellitus |  | 25,623 5,621 | 38.9% 33.0% | <0.001 | 0.122 |
|  |  | 1 2 | E65-E68 | Obesity |  | 16,984 3,772 | 25.8% 22.2% | <0.001 | 0.085 |
|  |  | 1 2 | N18 | Chronic kidney disease |  | 17,107 3,548 | 26.0% 20.8% | <0.001 | 0.121 |
|  |  | 1 2 | I63 | Cerebral infarction |  | 41,309 9,950 | 62.7% 58.5% | <0.001 | 0.087 |
|  |  | 1 2 | I73.9 | Peripheral arterial disease |  | 9,258 1,967 | 14.0% 11.6% | <0.001 | 0.075 |
|  |  | 1 2 | J18 | Pneumonia |  | 14,743 2,639 | 22.4% 15.5% | <0.001 | 0.176 |
|  |  | 1 2 | A41 | Sepsis |  | 11,762 1,463 | 17.8% 8.6% | <0.001 | 0.276 |
|  |  | 1 2 | M30-M36 | Systemic connective tissue disorders |  | 6,693 2,575 | 10.2% 15.1% | <0.001 | 0.150 |
|  | **Procedure** | | | | | | | | |
|  |  | Cohort | |  | Mean ± SD | Patients | % of Cohort | p-Value | Std diff. |
|  |  | 1 2 | 1013050 | Echocardiography Procedures |  | 24,812 5,196 | 37.6% 30.5% | <0.001 | 0.151 |
|  |  | 1 2 | 1013071 | Cardiac Catheterization Procedures |  | 4,832 894 | 7.3% 5.3% | <0.001 | 0.086 |
|  |  | 1 2 | 1019075 | Implantable, Insertable, and Wearable Cardiac Device Evaluations |  | 2,227 484 | 3.4% 2.8% | <0.001 | 0.031 |
|  |  | 1 2 | 1013109 | Intracardiac Electrophysiological Procedures/Studies |  | 797 162 | 1.2% 1.0% | 0.005 | 0.025 |
|  |  | 1 2 | 1013012 | Electrocardiogram, routine ECG with at least 12 leads |  | 37,327 8,575 | 56.6% 50.4% | <0.001 | 0.126 |
|  | **Medication** | | | | | | | | |
|  |  | Cohort | |  | Mean ± SD | Patients | % of Cohort | p-Value | Std diff. |
|  |  | 1 2 | CV350 | Lipid-lowering treatment |  | 33,947 7,940 | 51.5% 46.6% | <0.001 | 0.097 |
|  |  | 1 2 | CV100 | Beta blockers |  | 34,272 6,824 | 52.0% 40.1% | <0.001 | 0.241 |
|  |  | 1 2 | CV300 | Antiarrhythmics |  | 31,879 7,317 | 48.4% 43.0% | <0.001 | 0.108 |
|  |  | 1 2 | CV700 | Diuretics |  | 28,959 5,609 | 43.9% 32.9% | <0.001 | 0.227 |
|  |  | 1 2 | CV200 | Calcium channel blockers |  | 25,192 5,116 | 38.2% 30.1% | <0.001 | 0.173 |
|  |  | 1 2 | CV800 | ACE inhibitors |  | 20,425 4,461 | 31.0% 26.2% | <0.001 | 0.106 |
|  |  | 1 2 | CV805 | Angiotensin II inhibitor |  | 11,994 2,656 | 18.2% 15.6% | <0.001 | 0.069 |
|  |  | 1 2 | CV250 | Antianginals |  | 11,542 2,210 | 17.5% 13.0% | <0.001 | 0.126 |
|  |  | 1 2 | BL110 | Anticoagulant therapy |  | 37,986 7,757 | 57.6% 45.6% | <0.001 | 0.243 |
|  |  | 1 2 | BL117 | Antiplatelet therapy |  | 37,346 8,784 | 56.7% 51.6% | <0.001 | 0.102 |

**Supplementary Table 4.** Baseline characteristics comparison, before and after propensity score matching, of elderly patients with C-Reactive Protein levels 1-3 mg/L (cohort 1) and < 1 mg/L (cohort 2).

| **Cohort 1 (N = 12,586) and cohort 2 (N = 9,249) characteristics before propensity score matching** | | | | | | | | | |
| --- | --- | --- | --- | --- | --- | --- | --- | --- | --- |
|  | **Demographics** | | | | | | | | |
|  |  | Cohort | |  | Mean ± SD | Patients | % of Cohort | P-Value | Std diff. |
|  |  | 1 2 | Age | Age | 77.9 +/- 8.1 77.3 +/- 7.9 | 12,558 9,229 | 100% 100% | <0.001 | 0.086 |
|  |  | 1 2 | 2106-3 | White |  | 9,073 6,965 | 72.2% 75.5% | <0.001 | 0.073 |
|  |  | 1 2 | F | Female |  | 6,497 4,941 | 51.7% 53.5% | 0.008 | 0.036 |
|  |  | 1 2 | 2054-5 | Black or African American |  | 1,763 1,169 | 14.0% 12.7% | 0.003 | 0.040 |
|  |  | 1 2 | 2028-9 | Asian |  | 247 252 | 2.0% 2.7% | <0.001 | 0.050 |
|  | **Diagnosis** | | | | | | | | |
|  |  | Cohort | |  | Mean ± SD | Patients | % of Cohort | P-Value | Std diff. |
|  |  | 1 2 | I10-I16 | Arterial hypertension |  | 8,676 6,065 | 69.1% 65.7% | <0.001 | 0.072 |
|  |  | 1 2 | I20-I25 | Ischemic heart diseases |  | 4,482 3,230 | 35.7% 35.0% | 0.291 | 0.014 |
|  |  | 1 2 | I48 | Atrial fibrillation and flutter |  | 2,945 1,842 | 23.5% 20.0% | <0.001 | 0.085 |
|  |  | 1 2 | I50 | Heart failure |  | 2,717 1,780 | 21.6% 19.3% | <0.001 | 0.058 |
|  |  | 1 2 | I26-I28 | Pulmonary heart disease and diseases of pulmonary circulation |  | 1,418 962 | 11.3% 10.4% | 0.042 | 0.028 |
|  |  | 1 2 | E78 | Dyslipidaemia |  | 7,063 4,990 | 56.2% 54.1% | 0.001 | 0.044 |
|  |  | 1 2 | E08-E13 | Diabetes mellitus |  | 4,586 3,298 | 36.5% 35.7% | 0.234 | 0.016 |
|  |  | 1 2 | E65-E68 | Obesity |  | 2,567 1,798 | 20.4% 19.5% | 0.081 | 0.024 |
|  |  | 1 2 | N18 | Chronic kidney disease (CKD) |  | 2,862 2,214 | 22.8% 24.0% | 0.039 | 0.028 |
|  |  | 1 2 | I63 | Cerebral infarction |  | 8,146 5,553 | 64.9% 60.2% | <0.001 | 0.097 |
|  |  | 1 2 | I73.9 | Peripheral arterial disease |  | 1,692 1,282 | 13.5% 13.9% | 0.375 | 0.012 |
|  |  | 1 2 | J18 | Pneumonia |  | 2,035 1,494 | 16.2% 16.2% | 0.974 | <0.001 |
|  |  | 1 2 | A41 | Sepsis |  | 1,142 759 | 9.1% 8.2% | 0.025 | 0.031 |
|  |  | 1 2 | M30-M36 | Systemic connective tissue disorders |  | 1,372 1,192 | 10.9% 12.9% | <0.001 | 0.061 |
|  | **Procedure** | | | | | | | | |
|  |  | Cohort | |  | Mean ± SD | Patients | % of Cohort | P-Value | Std diff. |
|  |  | 1 2 | 1013050 | Echocardiography Procedures |  | 4,257 3,074 | 33.9% 33.3% | 0.362 | 0.013 |
|  |  | 1 2 | 1013071 | Cardiac Catheterization Procedures |  | 848 561 | 6.8% 6.1% | 0.046 | 0.028 |
|  |  | 1 2 | 1019075 | Implantable, Insertable, and Wearable Cardiac Device Evaluations |  | 528 357 | 4.2% 3.9% | 0.214 | 0.017 |
|  |  | 1 2 | 1013109 | Intracardiac Electrophysiological Procedures/Studies |  | 165 85 | 1.3% 0.9% | 0.007 | 0.037 |
|  |  | 1 2 | 1013012 | Electrocardiogram, routine ECG with at least 12 leads |  | 6,783 4,907 | 54.0% 53.2% | 0.217 | 0.017 |
|  | **Medication** | | | | | | | | |
|  |  | Cohort | |  | Mean ± SD | Patients | % of Cohort | P-Value | Std diff. |
|  |  | 1 2 | CV350 | Antilipemic agents |  | 7,408 5,148 | 59.0% 55.8% | <0.001 | 0.065 |
|  |  | 1 2 | CV100 | Beta blockers/related |  | 6,455 4,336 | 51.4% 47.0% | <0.001 | 0.088 |
|  |  | 1 2 | CV300 | Antiarrhythmics |  | 5,249 3,893 | 41.8% 42.2% | 0.570 | 0.008 |
|  |  | 1 2 | CV700 | Diuretics |  | 5,228 3,613 | 41.6% 39.1% | <0.001 | 0.051 |
|  |  | 1 2 | CV200 | Calcium channel blockers |  | 4,723 3,317 | 37.6% 35.9% | 0.012 | 0.035 |
|  |  | 1 2 | CV800 | ACE inhibitors |  | 4,044 2,805 | 32.2% 30.4% | 0.004 | 0.039 |
|  |  | 1 2 | CV805 | Angiotensin II inhibitor |  | 2,654 1,902 | 21.1% 20.6% | 0.346 | 0.013 |
|  |  | 1 2 | CV250 | Antianginals |  | 2,089 1,450 | 16.6% 15.7% | 0.068 | 0.025 |
|  |  | 1 2 | BL110 | Anticoagulant therapy |  | 6,579 4,475 | 52.4% 48.5% | <0.001 | 0.078 |
|  |  | 1 2 | BL117 | Antiplatelet therapy |  | 7,509 5,307 | 59.8% 57.5% | 0.001 | 0.047 |
| **Cohort 1 (N = 9,122) and cohort 2 (N = 9,122) characteristics after propensity score matching** | | | | | | | | | |
|  | **Demographics** | | | | | | | | |
|  |  | Cohort | |  | Mean ± SD | Patients | % of Cohort | P-Value | Std diff. |
|  |  | 1 2 | Age | Age | 77.2 +/- 8.1 77.3 +/- 7.9 | 9,122 9,122 | 100% 100% | 0.522 | 0.009 |
|  |  | 1 2 | 2106-3 | White |  | 6,871 6,883 | 75.3% 75.5% | 0.837 | 0.003 |
|  |  | 1 2 | F | Female |  | 4,839 4,856 | 53.0% 53.2% | 0.801 | 0.004 |
|  |  | 1 2 | 2054-5 | Black or African American |  | 1,170 1,164 | 12.8% 12.8% | 0.894 | 0.002 |
|  |  | 1 2 | 2028-9 | Asian |  | 227 232 | 2.5% 2.5% | 0.813 | 0.003 |
|  | **Diagnosis** | | | | | | | | |
|  |  | Cohort | |  | Mean ± SD | Patients | % of Cohort | P-Value | Std diff. |
|  |  | 1 2 | I10-I16 | Arterial hypertension |  | 5,971 6,011 | 65.5% 65.9% | 0.533 | 0.009 |
|  |  | 1 2 | I20-I25 | Ischemic heart diseases |  | 3,198 3,188 | 35.1% 34.9% | 0.877 | 0.002 |
|  |  | 1 2 | I48 | Atrial fibrillation and flutter |  | 1,865 1,839 | 20.4% 20.2% | 0.632 | 0.007 |
|  |  | 1 2 | I50 | Heart failure |  | 1,773 1,771 | 19.4% 19.4% | 0.970 | 0.001 |
|  |  | 1 2 | I26-I28 | Pulmonary heart disease and diseases of pulmonary circulation |  | 948 955 | 10.4% 10.5% | 0.865 | 0.003 |
|  |  | 1 2 | E78 | Dyslipidaemia |  | 4,934 4,939 | 54.1% 54.1% | 0.941 | 0.001 |
|  |  | 1 2 | E08-E13 | Diabetes mellitus |  | 3,238 3,259 | 35.5% 35.7% | 0.745 | 0.005 |
|  |  | 1 2 | E65-E68 | Obesity |  | 1,781 1,779 | 19.5% 19.5% | 0.970 | 0.001 |
|  |  | 1 2 | N18 | Chronic kidney disease (CKD) |  | 2,181 2,163 | 23.9% 23.7% | 0.754 | 0.005 |
|  |  | 1 2 | I63 | Cerebral infarction |  | 5,531 5,516 | 60.6% 60.5% | 0.820 | 0.003 |
|  |  | 1 2 | I73.9 | Peripheral arterial disease |  | 1,263 1,259 | 13.8% 13.8% | 0.932 | 0.001 |
|  |  | 1 2 | J18 | Pneumonia |  | 1,460 1,471 | 16.0% 16.1% | 0.824 | 0.003 |
|  |  | 1 2 | A41 | Sepsis |  | 767 758 | 8.4% 8.3% | 0.810 | 0.004 |
|  |  | 1 2 | M30-M36 | Systemic connective tissue disorders |  | 1,149 1,147 | 12.6% 12.6% | 0.964 | 0.001 |
|  | **Procedure** | | | | | | | | |
|  |  | Cohort | |  | Mean ± SD | Patients | % of Cohort | P-Value | Std diff. |
|  |  | 1 2 | 1013050 | Echocardiography Procedures |  | 3,058 3,033 | 33.5% 33.2% | 0.695 | 0.006 |
|  |  | 1 2 | 1013071 | Cardiac Catheterization Procedures |  | 578 559 | 6.3% 6.1% | 0.561 | 0.009 |
|  |  | 1 2 | 1019075 | Implantable, Insertable, and Wearable Cardiac Device Evaluations |  | 363 356 | 4.0% 3.9% | 0.790 | 0.004 |
|  |  | 1 2 | 1013109 | Intracardiac Electrophysiological Procedures/Studies |  | 86 84 | 0.9% 0.9% | 0.878 | 0.002 |
|  |  | 1 2 | 1013012 | Electrocardiogram, routine ECG with at least 12 leads |  | 4,836 4,839 | 53.0% 53.0% | 0.965 | 0.001 |
|  | **Medication** | | | | | | | | |
|  |  | Cohort | |  | Mean ± SD | Patients | % of Cohort | P-Value | Std diff. |
|  |  | 1 2 | CV350 | Antilipemic agents |  | 5,120 5,108 | 56.1% 56.0% | 0.858 | 0.003 |
|  |  | 1 2 | CV100 | Beta blockers/related |  | 4,314 4,318 | 47.3% 47.3% | 0.953 | 0.001 |
|  |  | 1 2 | CV300 | Antiarrhythmics |  | 3,844 3,832 | 42.1% 42.0% | 0.857 | 0.003 |
|  |  | 1 2 | CV700 | Diuretics |  | 3,592 3,576 | 39.4% 39.2% | 0.808 | 0.004 |
|  |  | 1 2 | CV200 | Calcium channel blockers |  | 3,310 3,288 | 36.3% 36.0% | 0.735 | 0.005 |
|  |  | 1 2 | CV800 | ACE inhibitors |  | 2,786 2,781 | 30.5% 30.5% | 0.936 | 0.001 |
|  |  | 1 2 | CV805 | Angiotensin II inhibitor |  | 1,883 1,885 | 20.6% 20.7% | 0.971 | 0.001 |
|  |  | 1 2 | CV250 | Antianginals |  | 1,461 1,441 | 16.0% 15.8% | 0.686 | 0.006 |
|  |  | 1 2 | BL110 | Anticoagulant therapy |  | 4,457 4,440 | 48.9% 48.7% | 0.801 | 0.004 |
|  |  | 1 2 | BL117 | Antiplatelet therapy |  | 5,237 5,249 | 57.4% 57.5% | 0.857 | 0.003 |

**Supplementary Table 5**. Baseline characteristics comparison, before and after propensity score matching, of elderly patients with C-Reactive Protein levels >3 mg/L (cohort 1) and < 1 mg/L (cohort 2).

| **Cohort 1 (N = 41,657) and cohort 2 (N = 9,277) characteristics before propensity score matching** | | | | | | | | | |
| --- | --- | --- | --- | --- | --- | --- | --- | --- | --- |
|  | **Demographics** | | | | | | | | |
|  |  | Cohort | |  | Mean ± SD | Patients | % of Cohort | P-Value | Std diff. |
|  |  | 1 2 | Age | Age | 78.2 +/- 8.1 77.3 +/- 7.9 | 41,524 9,257 | 100% 100% | <0.001 | 0.120 |
|  |  | 1 2 | 2106-3 | White |  | 28,779 6,965 | 69.3% 75.2% | <0.001 | 0.133 |
|  |  | 1 2 | F | Female |  | 20,706 4,957 | 49.9% 53.5% | <0.001 | 0.074 |
|  |  | 1 2 | 2054-5 | Black or African American |  | 6,726 1,169 | 16.2% 12.6% | <0.001 | 0.102 |
|  |  | 1 2 | 2028-9 | Asian |  | 595 252 | 1.4% 2.7% | <0.001 | 0.090 |
|  | **Diagnosis** | | | | | | | | |
|  |  | Cohort | |  | Mean ± SD | Patients | % of Cohort | P-Value | Std diff. |
|  |  | 1 2 | I10-I16 | Arterial hypertension |  | 30,333 6,068 | 73.0% 65.6% | <0.001 | 0.163 |
|  |  | 1 2 | I20-I25 | Ischemic heart diseases |  | 17,153 3,230 | 41.3% 34.9% | <0.001 | 0.132 |
|  |  | 1 2 | I48 | Atrial fibrillation and flutter |  | 12,127 1,842 | 29.2% 19.9% | <0.001 | 0.218 |
|  |  | 1 2 | I50 | Heart failure |  | 12,241 1,780 | 29.5% 19.2% | <0.001 | 0.241 |
|  |  | 1 2 | I26-I28 | Pulmonary heart disease and diseases of pulmonary circulation |  | 6,340 962 | 15.3% 10.4% | <0.001 | 0.146 |
|  |  | 1 2 | E78 | Dyslipidaemia |  | 22,992 4,990 | 55.4% 53.9% | 0.010 | 0.029 |
|  |  | 1 2 | E08-E13 | Diabetes mellitus |  | 16,982 3,298 | 40.9% 35.6% | <0.001 | 0.109 |
|  |  | 1 2 | E65-E68 | Obesity |  | 9,596 1,798 | 23.1% 19.4% | <0.001 | 0.090 |
|  |  | 1 2 | N18 | Chronic kidney disease (CKD) |  | 12,211 2,215 | 29.4% 23.9% | <0.001 | 0.124 |
|  |  | 1 2 | I63 | Cerebral infarction |  | 26,986 5,563 | 65.0% 60.1% | <0.001 | 0.101 |
|  |  | 1 2 | I73.9 | Peripheral arterial disease |  | 6,631 1,282 | 16.0% 13.8% | <0.001 | 0.060 |
|  |  | 1 2 | J18 | Pneumonia |  | 9,933 1,495 | 23.9% 16.1% | <0.001 | 0.195 |
|  |  | 1 2 | A41 | Sepsis |  | 7,373 759 | 17.8% 8.2% | <0.001 | 0.287 |
|  |  | 1 2 | M30-M36 | Systemic connective tissue disorders |  | 3,657 1,193 | 8.8% 12.9% | <0.001 | 0.132 |
|  | **Procedure** | | | | | | | | |
|  |  | Cohort | |  | Mean ± SD | Patients | % of Cohort | P-Value | Std diff. |
|  |  | 1 2 | 1013050 | Echocardiography Procedures |  | 16,696 3,074 | 40.2% 33.2% | <0.001 | 0.146 |
|  |  | 1 2 | 1013071 | Cardiac Catheterization Procedures |  | 3,207 561 | 7.7% 6.1% | <0.001 | 0.066 |
|  |  | 1 2 | 1019075 | Implantable, Insertable, and Wearable Cardiac Device Evaluations |  | 1,741 357 | 4.2% 3.9% | 0.142 | 0.017 |
|  |  | 1 2 | 1013109 | Intracardiac Electrophysiological Procedures/Studies |  | 572 85 | 1.4% 0.9% | <0.001 | 0.043 |
|  |  | 1 2 | 1013012 | Electrocardiogram, routine ECG with at least 12 leads |  | 24,622 4,907 | 59.3% 53.0% | <0.001 | 0.127 |
|  | **Medication** | | | | | | | | |
|  |  | Cohort | |  | Mean ± SD | Patients | % of Cohort | P-Value | Std diff. |
|  |  | 1 2 | CV350 | Antilipemic agents |  | 24,213 5,159 | 58.3% 55.7% | <0.001 | 0.052 |
|  |  | 1 2 | CV100 | Beta blockers/related |  | 23,701 4,341 | 57.1% 46.9% | <0.001 | 0.205 |
|  |  | 1 2 | CV300 | Antiarrhythmics |  | 20,053 3,894 | 48.3% 42.1% | <0.001 | 0.125 |
|  |  | 1 2 | CV700 | Diuretics |  | 20,071 3,615 | 48.3% 39.1% | <0.001 | 0.188 |
|  |  | 1 2 | CV200 | Calcium channel blockers |  | 17,698 3,323 | 42.6% 35.9% | <0.001 | 0.138 |
|  |  | 1 2 | CV800 | ACE inhibitors |  | 13,915 2,814 | 33.5% 30.4% | <0.001 | 0.067 |
|  |  | 1 2 | CV805 | Angiotensin II inhibitor |  | 8,950 1,905 | 21.6% 20.6% | 0.039 | 0.024 |
|  |  | 1 2 | CV250 | Antianginals |  | 8,156 1,451 | 19.6% 15.7% | <0.001 | 0.104 |
|  |  | 1 2 | BL110 | Anticoagulant therapy |  | 25,028 4,488 | 60.3% 48.5% | <0.001 | 0.238 |
|  |  | 1 2 | BL117 | Antiplatelet therapy |  | 25,549 5,319 | 61.5% 57.5% | <0.001 | 0.083 |
| **Cohort 1 (N = 9,244) and cohort 2 (N = 9,244) characteristics after propensity score matching** | | | | | | | | | |
|  | **Demographics** | | | | | | | | |
|  |  | Cohort | |  | Mean ± SD | Patients | % of Cohort | P-Value | Std diff. |
|  |  | 1 2 | Age | Age | 77.3 +/- 8.1 77.3 +/- 7.9 | 9,244 9,244 | 100% 100% | 0.504 | 0.010 |
|  |  | 1 2 | 2106-3 | White |  | 6,984 6,957 | 75.6% 75.3% | 0.645 | 0.007 |
|  |  | 1 2 | F | Female |  | 4,969 4,944 | 53.8% 53.5% | 0.712 | 0.005 |
|  |  | 1 2 | 2054-5 | Black or African American |  | 1,160 1,169 | 12.5% 12.6% | 0.842 | 0.003 |
|  |  | 1 2 | 2028-9 | Asian |  | 232 247 | 2.5% 2.7% | 0.487 | 0.010 |
|  | **Diagnosis** | | | | | | | | |
|  |  | Cohort | |  | Mean ± SD | Patients | % of Cohort | P-Value | Std diff. |
|  |  | 1 2 | I10-I16 | Arterial hypertension |  | 6,104 6,063 | 66.0% 65.6% | 0.525 | 0.009 |
|  |  | 1 2 | I20-I25 | Ischemic heart diseases |  | 3,218 3,226 | 34.8% 34.9% | 0.902 | 0.002 |
|  |  | 1 2 | I48 | Atrial fibrillation and flutter |  | 1,835 1,842 | 19.9% 19.9% | 0.897 | 0.002 |
|  |  | 1 2 | I50 | Heart failure |  | 1,739 1,780 | 18.8% 19.3% | 0.442 | 0.011 |
|  |  | 1 2 | I26-I28 | Pulmonary heart disease and diseases of pulmonary circulation |  | 962 962 | 10.4% 10.4% | 1 | <0.001 |
|  |  | 1 2 | E78 | Dyslipidaemia |  | 5,029 4,980 | 54.4% 53.9% | 0.470 | 0.011 |
|  |  | 1 2 | E08-E13 | Diabetes mellitus |  | 3,303 3,293 | 35.7% 35.6% | 0.878 | 0.002 |
|  |  | 1 2 | E65-E68 | Obesity |  | 1,829 1,797 | 19.8% 19.4% | 0.553 | 0.009 |
|  |  | 1 2 | N18 | Chronic kidney disease (CKD) |  | 2,203 2,209 | 23.8% 23.9% | 0.918 | 0.002 |
|  |  | 1 2 | I63 | Cerebral infarction |  | 5,564 5,555 | 60.2% 60.1% | 0.892 | 0.002 |
|  |  | 1 2 | I73.9 | Peripheral arterial disease |  | 1,262 1,281 | 13.7% 13.9% | 0.685 | 0.006 |
|  |  | 1 2 | J18 | Pneumonia |  | 1,534 1,494 | 16.6% 16.2% | 0.427 | 0.012 |
|  |  | 1 2 | A41 | Sepsis |  | 759 759 | 8.2% 8.2% | 1 | <0.001 |
|  |  | 1 2 | M30-M36 | Systemic connective tissue disorders |  | 1,223 1,182 | 13.2% 12.8% | 0.370 | 0.013 |
|  | **Procedure** | | | | | | | | |
|  |  | Cohort | |  | Mean ± SD | Patients | % of Cohort | P-Value | Std diff. |
|  |  | 1 2 | 1013050 | Echocardiography Procedures |  | 3,084 3,072 | 33.4% 33.2% | 0.851 | 0.003 |
|  |  | 1 2 | 1013071 | Cardiac Catheterization Procedures |  | 528 561 | 5.7% 6.1% | 0.303 | 0.015 |
|  |  | 1 2 | 1019075 | Implantable, Insertable, and Wearable Cardiac Device Evaluations |  | 351 357 | 3.8% 3.9% | 0.818 | 0.003 |
|  |  | 1 2 | 1013109 | Intracardiac Electrophysiological Procedures/Studies |  | 83 85 | 0.9% 0.9% | 0.877 | 0.002 |
|  |  | 1 2 | 1013012 | Electrocardiogram, routine ECG with at least 12 leads |  | 4,825 4,901 | 52.2% 53.0% | 0.263 | 0.016 |
|  | **Medication** | | | | | | | | |
|  |  | Cohort | |  | Mean ± SD | Patients | % of Cohort | P-Value | Std diff. |
|  |  | 1 2 | CV350 | Antilipemic agents |  | 5,124 5,147 | 55.4% 55.7% | 0.734 | 0.005 |
|  |  | 1 2 | CV100 | Beta blockers/related |  | 4,404 4,339 | 47.6% 46.9% | 0.338 | 0.014 |
|  |  | 1 2 | CV300 | Antiarrhythmics |  | 3,922 3,885 | 42.4% 42.0% | 0.582 | 0.008 |
|  |  | 1 2 | CV700 | Diuretics |  | 3,625 3,613 | 39.2% 39.1% | 0.857 | 0.003 |
|  |  | 1 2 | CV200 | Calcium channel blockers |  | 3,335 3,321 | 36.1% 35.9% | 0.830 | 0.003 |
|  |  | 1 2 | CV800 | ACE inhibitors |  | 2,814 2,810 | 30.4% 30.4% | 0.949 | 0.001 |
|  |  | 1 2 | CV805 | Angiotensin II inhibitor |  | 1,922 1,900 | 20.8% 20.6% | 0.689 | 0.006 |
|  |  | 1 2 | CV250 | Antianginals |  | 1,445 1,451 | 15.6% 15.7% | 0.903 | 0.002 |
|  |  | 1 2 | BL110 | Anticoagulant therapy |  | 4,482 4,486 | 48.5% 48.5% | 0.953 | 0.001 |
|  |  | 1 2 | BL117 | Antiplatelet therapy |  | 5,303 5,308 | 57.4% 57.4% | 0.941 | 0.001 |

**Supplementary Table 6.** Baseline characteristics comparison, before and after propensity score matching, of patients without confounding factors with C-Reactive Protein levels 1-3 mg/L (cohort 1) and < 1 mg/L (cohort 2).

| **Cohort 1 (N = 8,458) and cohort 2 (N = 6,546) characteristics before propensity score matching** | | | | | | | | | |
| --- | --- | --- | --- | --- | --- | --- | --- | --- | --- |
|  | **Demographics** | | | | | | | | |
|  |  | Cohort | |  | Mean ± SD | Patients | % of Cohort | P-Value | Std diff. |
|  |  | 1 2 | Age | Age | 67.5 +/- 16.5 66.0 +/- 16.8 | 8,437 6,532 | 100% 100% | <0.001 | 0.090 |
|  |  | 1 2 | 2106-3 | White |  | 5,477 4,382 | 64.9% 67.1% | 0.006 | 0.046 |
|  |  | 1 2 | F | Female |  | 3,996 3,140 | 47.4% 48.1% | 0.390 | 0.014 |
|  |  | 1 2 | 2054-5 | Black or African American |  | 1,370 1,069 | 16.2% 16.4% | 0.834 | 0.003 |
|  |  | 1 2 | 2028-9 | Asian |  | 201 252 | 2.4% 3.9% | <0.001 | 0.085 |
|  | **Diagnosis** | | | | | | | | |
|  |  | Cohort | |  | Mean ± SD | Patients | % of Cohort | P-Value | Std diff. |
|  |  | 1 2 | I10-I16 | Arterial hypertension |  | 4,190 2,896 | 49.7% 44.3% | <0.001 | 0.107 |
|  |  | 1 2 | I20-I25 | Ischemic heart diseases |  | 1,623 1,141 | 19.2% 17.5% | 0.006 | 0.046 |
|  |  | 1 2 | I48 | Atrial fibrillation and flutter |  | 1,038 623 | 12.3% 9.5% | <0.001 | 0.089 |
|  |  | 1 2 | I50 | Heart failure |  | 782 495 | 9.3% 7.6% | <0.001 | 0.061 |
|  |  | 1 2 | I26-I28 | Pulmonary heart disease and diseases of pulmonary circulation |  | 355 216 | 4.2% 3.3% | 0.004 | 0.047 |
|  |  | 1 2 | E78 | Dyslipidaemia |  | 3,226 2,251 | 38.2% 34.5% | <0.001 | 0.079 |
|  |  | 1 2 | E08-E13 | Diabetes mellitus |  | 1,924 1,322 | 22.8% 20.2% | <0.001 | 0.062 |
|  |  | 1 2 | E65-E68 | Obesity |  | 1,078 727 | 12.8% 11.1% | 0.002 | 0.051 |
|  |  | 1 2 | N18 | Chronic kidney disease (CKD) |  | 813 602 | 9.6% 9.2% | 0.384 | 0.014 |
|  |  | 1 2 | I63 | Cerebral infarction |  | 4,673 3,380 | 55.4% 51.7% | <0.001 | 0.073 |
|  |  | 1 2 | I73.9 | Peripheral arterial disease |  | 395 293 | 4.7% 4.5% | 0.570 | 0.009 |
|  | **Procedure** | | | | | | | | |
|  |  | Cohort | |  | Mean ± SD | Patients | % of Cohort | P-Value | Std diff. |
|  |  | 1 2 | 1013050 | Echocardiography Procedures |  | 1,631 1,246 | 19.3% 19.1% | 0.693 | 0.007 |
|  |  | 1 2 | 1013071 | Cardiac Catheterization Procedures |  | 278 178 | 3.3% 2.7% | 0.044 | 0.033 |
|  |  | 1 2 | 1019075 | Implantable, Insertable, and Wearable Cardiac Device Evaluations |  | 173 107 | 2.1% 1.6% | 0.065 | 0.031 |
|  |  | 1 2 | 1013109 | Intracardiac Electrophysiological Procedures/Studies |  | 57 30 | 0.7% 0.5% | 0.084 | 0.029 |
|  |  | 1 2 | 1013012 | Electrocardiogram, routine ECG with at least 12 leads |  | 3,177 2,419 | 37.7% 37.0% | 0.435 | 0.013 |
|  | **Medication** | | | | | | | | |
|  |  | Cohort | |  | Mean ± SD | Patients | % of Cohort | P-Value | Std diff. |
|  |  | 1 2 | CV350 | Antilipemic agents |  | 3,576 2,513 | 42.4% 38.5% | <0.001 | 0.080 |
|  |  | 1 2 | CV100 | Beta blockers/related |  | 2,888 1,999 | 34.2% 30.6% | <0.001 | 0.078 |
|  |  | 1 2 | CV300 | Antiarrhythmics |  | 1,893 1,518 | 22.4% 23.2% | 0.246 | 0.019 |
|  |  | 1 2 | CV700 | Diuretics |  | 1,976 1,409 | 23.4% 21.6% | 0.007 | 0.044 |
|  |  | 1 2 | CV200 | Calcium channel blockers |  | 2,039 1,429 | 24.2% 21.9% | 0.001 | 0.054 |
|  |  | 1 2 | CV800 | ACE inhibitors |  | 1,864 1,314 | 22.1% 20.1% | 0.003 | 0.048 |
|  |  | 1 2 | CV805 | Angiotensin II inhibitor |  | 1,080 738 | 12.8% 11.3% | 0.005 | 0.046 |
|  |  | 1 2 | CV250 | Antianginals |  | 777 491 | 9.2% 7.5% | <0.001 | 0.061 |
|  |  | 1 2 | BL110 | Anticoagulant therapy |  | 3,052 2,180 | 36.2% 33.4% | <0.001 | 0.059 |
|  |  | 1 2 | BL117 | Antiplatelet therapy |  | 3,717 2,751 | 44.1% 42.1% | 0.017 | 0.039 |
| **Cohort 1 (N = 6,387) and cohort 2 (N = 6,387) characteristics after propensity score matching** | | | | | | | | | |
|  | **Demographics** | | | | | | | | |
|  |  | Cohort | |  | Mean ± SD | Patients | % of Cohort | P-Value | Std diff. |
|  |  | 1 2 | Age | Age | 66.3 +/- 16.9 66.3 +/- 16.7 | 6,387 6,387 | 100% 100% | 0.862 | 0.003 |
|  |  | 1 2 | 2106-3 | White |  | 4,310 4,312 | 67.5% 67.5% | 0.970 | 0.001 |
|  |  | 1 2 | F | Female |  | 3,061 3,071 | 47.9% 48.1% | 0.859 | 0.003 |
|  |  | 1 2 | 2054-5 | Black or African American |  | 1,038 1,055 | 16.3% 16.5% | 0.684 | 0.007 |
|  |  | 1 2 | 2028-9 | Asian |  | 197 191 | 3.1% 3.0% | 0.757 | 0.005 |
|  | **Diagnosis** | | | | | | | | |
|  |  | Cohort | |  | Mean ± SD | Patients | % of Cohort | P-Value | Std diff. |
|  |  | 1 2 | I10-I16 | Arterial hypertension |  | 2,839 2,859 | 44.4% 44.8% | 0.722 | 0.006 |
|  |  | 1 2 | I20-I25 | Ischemic heart diseases |  | 1,103 1,120 | 17.3% 17.5% | 0.692 | 0.007 |
|  |  | 1 2 | I48 | Atrial fibrillation and flutter |  | 650 619 | 10.2% 9.7% | 0.359 | 0.016 |
|  |  | 1 2 | I50 | Heart failure |  | 490 490 | 7.7% 7.7% | 1 | <0.001 |
|  |  | 1 2 | I26-I28 | Pulmonary heart disease and diseases of pulmonary circulation |  | 220 213 | 3.4% 3.3% | 0.732 | 0.006 |
|  |  | 1 2 | E78 | Dyslipidaemia |  | 2,209 2,210 | 34.6% 34.6% | 0.985 | <0.001 |
|  |  | 1 2 | E08-E13 | Diabetes mellitus |  | 1,290 1,302 | 20.2% 20.4% | 0.792 | 0.005 |
|  |  | 1 2 | E65-E68 | Obesity |  | 717 716 | 11.2% 11.2% | 0.978 | <0.001 |
|  |  | 1 2 | N18 | Chronic kidney disease (CKD) |  | 583 586 | 9.1% 9.2% | 0.927 | 0.002 |
|  |  | 1 2 | I63 | Cerebral infarction |  | 3,304 3,331 | 51.7% 52.2% | 0.633 | 0.008 |
|  |  | 1 2 | I73.9 | Peripheral arterial disease |  | 280 287 | 4.4% 4.5% | 0.764 | 0.005 |
|  | **Procedure** | | | | | | | | |
|  |  | Cohort | |  | Mean ± SD | Patients | % of Cohort | P-Value | Std diff. |
|  |  | 1 2 | 1013050 | Echocardiography Procedures |  | 1,209 1,211 | 18.9% 19.0% | 0.964 | 0.001 |
|  |  | 1 2 | 1013071 | Cardiac Catheterization Procedures |  | 187 177 | 2.9% 2.8% | 0.595 | 0.009 |
|  |  | 1 2 | 1019075 | Implantable, Insertable, and Wearable Cardiac Device Evaluations |  | 112 106 | 1.8% 1.7% | 0.682 | 0.007 |
|  |  | 1 2 | 1013109 | Intracardiac Electrophysiological Procedures/Studies |  | 32 30 | 0.5% 0.5% | 0.799 | 0.005 |
|  |  | 1 2 | 1013012 | Electrocardiogram, routine ECG with at least 12 leads |  | 2,347 2,350 | 36.7% 36.8% | 0.956 | 0.001 |
|  | **Medication** | | | | | | | | |
|  |  | Cohort | |  | Mean ± SD | Patients | % of Cohort | P-Value | Std diff. |
|  |  | 1 2 | CV350 | Antilipemic agents |  | 2,454 2,475 | 38.4% 38.8% | 0.703 | 0.007 |
|  |  | 1 2 | CV100 | Beta blockers/related |  | 1,965 1,971 | 30.8% 30.9% | 0.908 | 0.002 |
|  |  | 1 2 | CV300 | Antiarrhythmics |  | 1,455 1,452 | 22.8% 22.7% | 0.950 | 0.001 |
|  |  | 1 2 | CV700 | Diuretics |  | 1,387 1,381 | 21.7% 21.6% | 0.897 | 0.002 |
|  |  | 1 2 | CV200 | Calcium channel blockers |  | 1,409 1,412 | 22.1% 22.1% | 0.949 | 0.001 |
|  |  | 1 2 | CV800 | ACE inhibitors |  | 1,295 1,295 | 20.3% 20.3% | 1 | <0.001 |
|  |  | 1 2 | CV805 | Angiotensin ii inhibitor |  | 738 728 | 11.6% 11.4% | 0.781 | 0.005 |
|  |  | 1 2 | CV250 | Antianginals |  | 490 489 | 7.7% 7.7% | 0.973 | 0.001 |
|  |  | 1 2 | BL110 | Anticoagulant therapy |  | 2,113 2,147 | 33.1% 33.6% | 0.523 | 0.011 |
|  |  | 1 2 | BL117 | Antiplatelet therapy |  | 2,717 2,690 | 42.5% 42.1% | 0.629 | 0.009 |

**Supplementary Table 7.** Baseline characteristics comparison, before and after propensity score matching, of patients without confounding factors with C-Reactive Protein levels >3 mg/L (cohort 1) and < 1 mg/L (cohort 2).

| **Cohort 1 (N = 17,640) and cohort 2 (N = 6,546) characteristics before propensity score matching** | | | | | | | | | |
| --- | --- | --- | --- | --- | --- | --- | --- | --- | --- |
|  | **Demographics** | | | | | | | | |
|  |  | Cohort | |  | Mean ± SD | Patients | % of Cohort | P-Value | Std diff. |
|  |  | 1 2 | Age | Age | 69.0 +/- 16.1 66.0 +/- 16.8 | 17,558 6,532 | 100% 100% | <0.001 | 0.179 |
|  |  | 1 2 | 2106-3 | White |  | 10,933 4,382 | 62.3% 67.1% | <0.001 | 0.101 |
|  |  | 1 2 | F | Female |  | 8,759 3,140 | 49.9% 48.1% | 0.012 | 0.036 |
|  |  | 1 2 | 2054-5 | Black or African American |  | 3,222 1,069 | 18.4% 16.4% | <0.001 | 0.052 |
|  |  | 1 2 | 2028-9 | Asian |  | 258 252 | 1.5% 3.9% | <0.001 | 0.149 |
|  | **Diagnosis** | | | | | | | | |
|  |  | Cohort | |  | Mean ± SD | Patients | % of Cohort | P-Value | Std diff. |
|  |  | 1 2 | I10-I16 | Arterial hypertension |  | 9,736 2,896 | 55.5% 44.3% | <0.001 | 0.224 |
|  |  | 1 2 | I20-I25 | Ischemic heart diseases |  | 4,009 1,141 | 22.8% 17.5% | <0.001 | 0.134 |
|  |  | 1 2 | I48 | Atrial fibrillation and flutter |  | 2,766 623 | 15.8% 9.5% | <0.001 | 0.188 |
|  |  | 1 2 | I50 | Heart failure |  | 2,533 495 | 14.4% 7.6% | <0.001 | 0.220 |
|  |  | 1 2 | I26-I28 | Pulmonary heart disease and diseases of pulmonary circulation |  | 1,124 216 | 6.4% 3.3% | <0.001 | 0.144 |
|  |  | 1 2 | E78 | Dyslipidaemia |  | 6,941 2,251 | 39.5% 34.5% | <0.001 | 0.105 |
|  |  | 1 2 | E08-E13 | Diabetes mellitus |  | 4,769 1,322 | 27.2% 20.2% | <0.001 | 0.163 |
|  |  | 1 2 | E65-E68 | Obesity |  | 2,735 727 | 15.6% 11.1% | <0.001 | 0.131 |
|  |  | 1 2 | N18 | Chronic kidney disease (CKD) |  | 2,404 602 | 13.7% 9.2% | <0.001 | 0.141 |
|  |  | 1 2 | I63 | Cerebral infarction |  | 10,148 3,380 | 57.8% 51.7% | <0.001 | 0.122 |
|  |  | 1 2 | I73.9 | Peripheral arterial disease |  | 1,090 293 | 6.2% 4.5% | <0.001 | 0.077 |
|  | **Procedure** | | | | | | | | |
|  |  | Cohort | |  | Mean ± SD | Patients | % of Cohort | P-Value | Std diff. |
|  |  | 1 2 | 1013050 | Echocardiography Procedures |  | 3,800 1,246 | 21.6% 19.1% | <0.001 | 0.064 |
|  |  | 1 2 | 1013071 | Cardiac Catheterization Procedures |  | 619 178 | 3.5% 2.7% | 0.002 | 0.046 |
|  |  | 1 2 | 1019075 | Implantable, Insertable, and Wearable Cardiac Device Evaluations |  | 326 107 | 1.9% 1.6% | 0.256 | 0.017 |
|  |  | 1 2 | 1013109 | Intracardiac Electrophysiological Procedures/Studies |  | 124 30 | 0.7% 0.5% | 0.033 | 0.032 |
|  |  | 1 2 | 1013012 | Electrocardiogram, routine ECG with at least 12 leads |  | 7,084 2,419 | 40.3% 37.0% | <0.001 | 0.068 |
|  | **Medication** | | | | | | | | |
|  |  | Cohort | |  | Mean ± SD | Patients | % of Cohort | P-Value | Std diff. |
|  |  | 1 2 | CV350 | Antilipemic agents |  | 7,517 2,513 | 42.8% 38.5% | <0.001 | 0.088 |
|  |  | 1 2 | CV100 | Beta blockers/related |  | 6,938 1,999 | 39.5% 30.6% | <0.001 | 0.188 |
|  |  | 1 2 | CV300 | Antiarrhythmics |  | 4,315 1,518 | 24.6% 23.2% | 0.031 | 0.031 |
|  |  | 1 2 | CV700 | Diuretics |  | 4,974 1,409 | 28.3% 21.6% | <0.001 | 0.157 |
|  |  | 1 2 | CV200 | Calcium channel blockers |  | 4,880 1,429 | 27.8% 21.9% | <0.001 | 0.137 |
|  |  | 1 2 | CV800 | ACE inhibitors |  | 4,166 1,314 | 23.7% 20.1% | <0.001 | 0.087 |
|  |  | 1 2 | CV805 | Angiotensin II inhibitor |  | 2,423 738 | 13.8% 11.3% | <0.001 | 0.076 |
|  |  | 1 2 | CV250 | Antianginals |  | 1,869 491 | 10.6% 7.5% | <0.001 | 0.109 |
|  |  | 1 2 | BL110 | Anticoagulant therapy |  | 7,079 2,180 | 40.3% 33.4% | <0.001 | 0.144 |
|  |  | 1 2 | BL117 | Antiplatelet therapy |  | 7,946 2,751 | 45.3% 42.1% | <0.001 | 0.063 |
| **Cohort 1 (N = 6,477) and cohort 2 (N = 6,477) characteristics after propensity score matching** | | | | | | | | | |
|  | **Demographics** | | | | | | | | |
|  |  | Cohort | |  | Mean ± SD | Patients | % of Cohort | P-Value | Std diff. |
|  |  | 1 2 | Age | Age | 66.3 +/- 17.1 66.1 +/- 16.8 | 6,477 6,477 | 100% 100% | 0.621 | 0.009 |
|  |  | 1 2 | 2106-3 | White |  | 4,435 4,377 | 68.5% 67.6% | 0.275 | 0.019 |
|  |  | 1 2 | F | Female |  | 3,172 3,119 | 49.0% 48.2% | 0.351 | 0.016 |
|  |  | 1 2 | 2054-5 | Black or African American |  | 1,029 1,069 | 15.9% 16.5% | 0.340 | 0.017 |
|  |  | 1 2 | 2028-9 | Asian |  | 209 202 | 3.2% 3.1% | 0.726 | 0.006 |
|  | **Diagnosis** | | | | | | | | |
|  |  | Cohort | |  | Mean ± SD | Patients | % of Cohort | P-Value | Std diff. |
|  |  | 1 2 | I10-I16 | Arterial hypertension |  | 2,806 2,886 | 43.3% 44.6% | 0.157 | 0.025 |
|  |  | 1 2 | I20-I25 | Ischemic heart diseases |  | 1,125 1,137 | 17.4% 17.6% | 0.781 | 0.005 |
|  |  | 1 2 | I48 | Atrial fibrillation and flutter |  | 589 623 | 9.1% 9.6% | 0.305 | 0.018 |
|  |  | 1 2 | I50 | Heart failure |  | 494 495 | 7.6% 7.6% | 0.974 | 0.001 |
|  |  | 1 2 | I26-I28 | Pulmonary heart disease and diseases of pulmonary circulation |  | 239 216 | 3.7% 3.3% | 0.272 | 0.019 |
|  |  | 1 2 | E78 | Dyslipidaemia |  | 2,216 2,237 | 34.2% 34.5% | 0.698 | 0.007 |
|  |  | 1 2 | E08-E13 | Diabetes mellitus |  | 1,332 1,315 | 20.6% 20.3% | 0.711 | 0.007 |
|  |  | 1 2 | E65-E68 | Obesity |  | 734 727 | 11.3% 11.2% | 0.846 | 0.003 |
|  |  | 1 2 | N18 | Chronic kidney disease (CKD) |  | 607 601 | 9.4% 9.3% | 0.856 | 0.003 |
|  |  | 1 2 | I63 | Cerebral infarction |  | 3,303 3,354 | 51.0% 51.8% | 0.370 | 0.016 |
|  |  | 1 2 | I73.9 | Peripheral arterial disease |  | 294 293 | 4.5% 4.5% | 0.966 | 0.001 |
|  | **Procedure** | | | | | | | | |
|  |  | Cohort | |  | Mean ± SD | Patients | % of Cohort | P-Value | Std diff. |
|  |  | 1 2 | 1013050 | Echocardiography Procedures |  | 1,210 1,235 | 18.7% 19.1% | 0.575 | 0.010 |
|  |  | 1 2 | 1013071 | Cardiac Catheterization Procedures |  | 181 177 | 2.8% 2.7% | 0.830 | 0.004 |
|  |  | 1 2 | 1019075 | Implantable, Insertable, and Wearable Cardiac Device Evaluations |  | 88 104 | 1.4% 1.6% | 0.245 | 0.020 |
|  |  | 1 2 | 1013109 | Intracardiac Electrophysiological Procedures/Studies |  | 26 30 | 0.4% 0.5% | 0.592 | 0.009 |
|  |  | 1 2 | 1013012 | Electrocardiogram, routine ECG with at least 12 leads |  | 2,303 2,402 | 35.6% 37.1% | 0.071 | 0.032 |
|  | **Medication** | | | | | | | | |
|  |  | Cohort | |  | Mean ± SD | Patients | % of Cohort | P-Value | Std diff. |
|  |  | 1 2 | CV350 | Antilipemic agents |  | 2,438 2,490 | 37.6% 38.4% | 0.347 | 0.017 |
|  |  | 1 2 | CV100 | Beta blockers/related |  | 1,970 1,992 | 30.4% 30.8% | 0.675 | 0.007 |
|  |  | 1 2 | CV300 | Antiarrhythmics |  | 1,459 1,501 | 22.5% 23.2% | 0.379 | 0.015 |
|  |  | 1 2 | CV700 | Diuretics |  | 1,390 1,404 | 21.5% 21.7% | 0.765 | 0.005 |
|  |  | 1 2 | CV200 | Calcium channel blockers |  | 1,419 1,424 | 21.9% 22.0% | 0.915 | 0.002 |
|  |  | 1 2 | CV800 | ACE inhibitors |  | 1,317 1,306 | 20.3% 20.2% | 0.810 | 0.004 |
|  |  | 1 2 | CV805 | Angiotensin II inhibitor |  | 738 733 | 11.4% 11.3% | 0.890 | 0.002 |
|  |  | 1 2 | CV250 | Antianginals |  | 478 489 | 7.4% 7.5% | 0.713 | 0.006 |
|  |  | 1 2 | BL110 | Anticoagulant therapy |  | 2,138 2,169 | 33.0% 33.5% | 0.563 | 0.010 |
|  |  | 1 2 | BL117 | Antiplatelet therapy |  | 2,650 2,727 | 40.9% 42.1% | 0.170 | 0.024 |
